# Open quantum dynamics for plant motions

**DOI:** 10.1038/s41598-022-07102-w

**Published:** 2022-02-23

**Authors:** Dorje C. Brody

**Affiliations:** 1 Department of Mathematics, University of Surrey, Guildford, GU2 7XH UK; 2 St Petersburg National Research University of Information Technologies, Mechanics and Optics, St Petersburg, Russia 197101

**Keywords:** Quantum mechanics, Plant sciences, Mathematics and computing

## Abstract

Stochastic Schrödinger equations that govern the dynamics of open quantum systems are given by the equations for signal processing. In particular, the Brownian motion that drives the wave function of the system does not represent noise, but provides purely the arrival of new information. Thus the wave function is guided by the optimal signal detection about the conditions of the environments under noisy observations. This behaviour is similar to biological systems that detect environmental cues, process this information, and adapt to them optimally by minimising uncertainties about the conditions of their environments. It is postulated that information-processing capability is a fundamental law of nature, and hence that models describing open quantum systems can equally be applied to biological systems to model their dynamics. For illustration, simple stochastic models are considered to capture heliotropic and gravitropic motions of plants. The advantage of such dynamical models is that they allow for the quantification of information processed by the plants. By considering the consequence of information erasure, it is argued that biological systems can process environmental signals relatively close to the Landauer limit of computation, and that loss of information must lie at the heart of ageing in biological systems.

## Introduction

Biological systems are constantly exposed to changing environments but manage to adapt to them by gathering information about the conditions of their surroundings and processing this information to arrive at best strategies. This ability to gather and process information about the surrounding environment does not require sophisticated organs like the brain of an animal. For instance, slime mould—a large brainless amoeba-like cell—can solve computationally difficult problems in a relatively short time^1,2^. When the root of a plant encounters another root, it can detect whether it is the root of the same plant or it belongs to another plant^3^. The parasitic plant Cuscuta (dodder) are capable of processing olfactory signals to identify host locations and distinguish plants of differing nutrient levels^4^. Or, recent experiments suggest that common bean plants can detect the existence of objects in their vicinities without physical contacts^5^. These, and countless other examples, beg the questions how basic biological systems such as a single cell are capable of processing and storing information, and how can one model the dynamics of biological systems resulting from their information processing capabilities.

The phenomenon of responding to the changing environment, however, is not restricted to biological systems. Take, for instance, a quantum system like an atom or even a single particle. When a quantum system is immersed in an open environment, its state responds to the conditions of the environment. The dynamics of the system can then be interpreted as the result of signal processing, for, as demonstrated below, the dynamical equation governing the wave function of the system (the stochastic unravelling of the Lindblad equation) is precisely the equation for the optimal signal detection. Hence a quantum system in an open environment behaves as if it is a micro information processor, just like an aggregate of quantum particles forming biological systems. From this point of view it is natural to enquire whether the dynamical equation for the evolution of the wave function in an open environment can be used to also describe behaviours of biological systems.

The purpose of this paper is to explore the hypothesis that signal processing capability is fundamental to laws of nature, applicable to a wide range of systems including both quantum and biological, and examine its consequences. Evidently there are innumerable—if not all—biological phenomena that may be addressed from the viewpoint of signal processing. For definiteness we shall focus on the motions of plants, in particular, the phenomena known as heliotropism or phototropism—the solar or light tracking of plants^6^, and gravitropism—the gravitational-field tracking of plant roots^7^. While scientific study of heliotropism and gravitropism goes a long way^8–10^, the understanding of the biokinematics as well as the purposes for heliotropism and gravitropism are only emerging in recent years^11–14^. Our purpose here is to characterise qualitatively the dynamical behaviours of plants that result from information processing by employing familiar models used in open quantum systems. This not only demonstrates how models of signal detection are universally applicable to capture behaviours of quantum as well as biological systems, but also provides a new tool to model, and hence to make predictions about the statistics, of plant motions. Specifically, for a qualitative modelling of light-tracking motions of plants we shall employ a stochastic Schrödinger equation for which the Lindblad operator is given by the position operator^15^, but for the Hamiltonian we take it to be simply the momentum operator. Borrowing techniques of signal processing, we derive an exact solution to the dynamical equation which describes the one-dimensional tracking of a deterministic motion. Similarly, for gravity-tracking motions of plant-root orientations we employ a stochastic Schrödinger equation where both the Lindblad operator and the Hamiltonian are given by the Pauli matrix, for which the exact solutions are known^16^.

The significance of the introduction of dynamical models for plant motions under uncertain environments—a concept hitherto missing in the literature—is that such models allow for the quantification of the information processed, as well as information erased. From Landauer’s principle^17^, then, erasure of information is accompanied by energy consumption and heat production. We believe that this information erasure process, resulting in the increase of entropy, must lie at the heart of ageing, or arrow of time, in biological systems. This point of view has been advocated in the past^18,19^, though the introduction of concrete dynamical models for describing the behaviours of biological systems in response to the changes of their environmental conditions under the influence of noise has hitherto been missing. In particular, our model provides a rough estimate for the amount of energy consumption in plants due to erasure of processed information. For instance, if a daisy flower were to process information at the Landauer limit, then our model predicts that the flowers will consume of order $10^{-2}$ eV of energy overnight per each information-bearing cell by erasing the processed information to track the sun during the day. In the case of circumnutation of bean plants, our estimates show that the erasure of information about the location of a climbing support requires about 1 eV of energy per each information-bearing cell at the Landauer limit. This indicates that inefficient information processing can be detrimental to their survival. The fact that most plants produce little heat, in particular, supports our hypothesis that they are able to process information at a level significantly closer to the Landauer limit than mechanical devices at our disposal.

## Signal processing quantum dynamics

We begin by establishing the relation between the stochastic Schrödinger equation associated with Lindbladean dynamics and signal processing. This connection has long been envisaged by Belavkin and others^20^, but was made explicit by Brody & Hughston^16,21,22^ as an effective tool for solving, and thus arriving at weak solutions to certain types of stochastic Schrödinger equations. To start, consider the following problem in signal processing. We have an unknown quantity of interest, e.g., a signal or a message, represented by the random variable *L*. Let us assume that *L* takes discrete values $\{l_i\}$ with the probabilities $\{p_i\}$. The true value of *L* is unknown to the observer, who merely receives information about the value of the signal *L* that is obscured by noise. Assuming that noise is additive and is modelled by a Brownian motion $\{B_t\}$, and that the signal is revealed in time at a constant rate $\sigma$, the noisy observation of the signal is characterised by the information process

1 $$\begin{aligned} \xi _t = \sigma L t + B_t. \end{aligned}$$

In the literature of signal detection, the signal-plus-noise time series $\{\xi _t\}$ such as the one defined here is called the observation process. However, because $\{\xi _t\}$ models the flow of information, while in the quantum context the term “observation” can have multiple meanings, we shall refer to $\{\xi _t\}$ as the information process. Note that if there is a large number of random additive contributions to noise, then the law of large numbers implies that it is reasonable to assume that additive noise is normally distributed, making Brownian motion a reasonable candidate to model noise. The best estimate of *L* that minimises the quadratic error, given the observed time series $\{\xi _s\}_{s\le t}$ up to time *t*, is given by the conditional expectation

2 $$\begin{aligned} \langle L\rangle _t = \sum _{i} l_i \, {\mathbb P}\left( L=l_i|\{\xi _s\}_{s\le t} \right) . \end{aligned}$$

The problem thus reduces to working out the conditional probability $\pi _{it} = {\mathbb P}(L=l_i|\{\xi _s\}_{s\le t})$ that $L=l_i$ given the observation. From the Markov property of the information process and the fact that $L=\lim _{t\rightarrow \infty }\xi _t/(\sigma t)$, the conditional probability reduces to a simpler expression $\pi _{it} = {\mathbb P}(L=l_i|\xi _t)$, which can easily be determined by use of the Bayes formula:

3 $$\begin{aligned} {\mathbb P}(L=l_i|\xi _t) = \frac{{\mathbb P}(L=l_i)\,\rho (\xi _t|L=l_i)}{\sum _j {\mathbb P}(L=l_j)\,\rho (\xi _t|L=l_j)} \, . \end{aligned}$$

Here $\rho (\xi _t|L=l_j)$ denotes the density function of $\xi _t$ conditional on the event that $L=l_j$. From ${\mathbb P}(L=l_i)=p_i$, and the fact that conditional on $L=l_i$ the random variable $\xi _t$ is normally distributed with mean $\sigma l_i t$ and variance *t*, we deduce that

4 $$\begin{aligned} \rho (\xi |L=l_i)=\frac{1}{\sqrt{2\pi t}} \exp \left( - \frac{(\xi -\sigma l_i t)^2}{2t}\right) , \end{aligned}$$

and hence, upon substitution, that

5 $$\begin{aligned} \pi _{it} = \frac{p_i\,\exp \left( \sigma l_i\, \xi _t - \frac{1}{2} \sigma ^2 l_i^2 t\right) }{\sum _j p_j\,\exp \left( \sigma l_j\, \xi _t - \frac{1}{2} \sigma ^2 l_j^2 t\right) } \, . \end{aligned}$$

We now consider taking the stochastic differential of the conditional probability process $\{\pi _{it}\}$. Because $\{\pi _{it}\}$ is a smooth function of the two variables $t,\xi _t$, by use of Ito’s formula

6 $$\begin{aligned} \mathrm{d}f(t,\xi _t) = {\dot{f}}(t,\xi _t) \, \mathrm{d}t + f'(t,\xi _t) \, \mathrm{d}\xi _t + \frac{1}{2}f''(t,\xi _t) \, \mathrm{d}t \end{aligned}$$

for any smooth function *f*(*t*, *x*) of two variables, where dot denotes differentiation with respect to *t* and dash denotes differentiation with respect to *x*, a calculation shows that

7 $$\begin{aligned} \mathrm{d}\pi _{it} = \sigma \pi _{it} \big ( l_i - \langle L\rangle _t \big ) \left( \mathrm{d}\xi _t - \sigma \langle L\rangle _t \, \mathrm{d}t \right) . \end{aligned}$$

Introducing the process $\{W_t\}$ according to

8 $$\begin{aligned} W_t = \xi _t - \sigma \int _0^t \langle L\rangle _s \, \mathrm{d}s , \end{aligned}$$

we can show that $\{W_t\}$ thus defined is a standard Brownian motion under the physical probability measure ${\mathbb P}$^21^. In signal detection, the process $\{W_t\}$ arising in this manner is called the innovations process^23^, and it reveals the arrival of new information. That is, the increment $\mathrm{d}\xi _t$ in the observed time series contains both new information as well as redundant known information about the signal *L*. Removing $\sigma \langle L\rangle _t \,\mathrm{d}t$ from this increment, we are left with only the new information that was not already known at time *t*. The fact that $\{W_t\}$ represents new information, however, follows only if $\langle L\rangle _t$ is the “best” estimate of *L* that minimises the uncertainty (conditional variance) of *L*, exhausting all the relevant information gathered up to time *t*. We shall return to the significance of this statement in open quantum systems shortly, but for now we conclude that the increments $\mathrm{d}\pi _{it} = \sigma \pi _{it} ( l_i - \langle L\rangle _t) \,\mathrm{d}W_t$ of the conditional probability process are proportional to the increments of the innovations process.

If we define $\phi _{it}=\sqrt{\pi _{it}}$ to be the square root probability, then another application of Ito’s formula shows that

9 $$\begin{aligned} \mathrm{d}\sqrt{\pi _{it}} = \frac{1}{2\sqrt{\pi _{it}}}\,\mathrm{d}\pi _{it} - \frac{1}{8\pi _{it}\sqrt{\pi _{it}}}\,(\mathrm{d}\pi _{it})^2 , \end{aligned}$$

and hence from $(\mathrm{d}\pi _{it})^2=\sigma ^2 \pi _{it}^2 (l_i-\langle L\rangle _t)^2\mathrm{d}t$ that

10 $$\begin{aligned} \mathrm{d}\phi _{it} = \textstyle {\frac{1}{2}} \sigma \big ( l_i - \langle L\rangle _t \big ) \, \phi _{it} \, \mathrm{d}W_t - \textstyle {\frac{1}{8}} \sigma ^2 \big ( l_i - \langle L\rangle _t \big )^2 \phi _{it} \, \mathrm{d}t. \end{aligned}$$

With this expression in mind, let us consider a Hilbert space ${\mathscr {H}}$ associated with a physical system, on which an operator ${\hat{L}}$ is defined, whose eigenvalues are $\{l_i\}$ and eigenstates are $\{|l_i\rangle \}$. Assume that the Hamiltonian ${\hat{H}}$ of the system, whose eigenvalues are $\{E_i\}$, commutes with ${\hat{L}}$, and that the eigenvalues of the two operators are not degenerate (the degenerate case can equally be treated^21^). For simplicity let the initial state of the system be given by a pure state $|\psi _0\rangle$, and let $p_i=|\langle l_i|\psi _0\rangle |^2$. Let the process $|\psi _t\rangle$ be given by

11 $$\begin{aligned} |\psi _t\rangle = \sum _i \phi _{it} \, \mathrm{e}^{\mathrm{i}(\theta _i-E_i t)}\, | l_i\rangle , \end{aligned}$$

where $\{\theta _i\}$ are arbitrary constant and $\phi _{it}$ satisfies (10). Then from the discussion above it should be evident, by taking the stochastic differential of (11), that $|\psi _t\rangle$ *is* the solution to the stochastic Schrödinger equation

12 $$\begin{aligned} \mathrm{d}|\psi _t\rangle = - \mathrm{i}{\hat{H}}\, |\psi _t\rangle \, \mathrm{d}t + \textstyle {\frac{1}{2}} \sigma \big ( {\hat{L}} - \langle {\hat{L}}\rangle _t \big ) \, |\psi _t\rangle \, \mathrm{d}W_t - \textstyle {\frac{1}{8}} \sigma ^2 \big ( {\hat{L}} - \langle {\hat{L}}\rangle _t \big )^2 |\psi _t\rangle \, \mathrm{d}t , \end{aligned}$$

where

13 $$\begin{aligned} \langle {\hat{L}}\rangle _t = \langle \psi _t|{\hat{L}}|\psi _t\rangle = \sum _{i} l_i \, {\mathbb P}\left( L=l_i|\xi _t\right) , \end{aligned}$$

and the constants $\{\theta _i\}$ are fixed by the relative phases of the initial condition $|\psi _0\rangle$. This is the method introduced to find solutions to the so-called energy-based stochastic Schrödinger equation for which ${\hat{L}}={\hat{H}}$, that is, when the Lindblad operator is given by the Hamiltonian itself^16^. But (12) is the dynamical equation for the wave function of the open quantum system that gives the stochastic unravelling of the corresponding Lindblad equation

14 $$\begin{aligned} \partial _t {\hat{\rho }} = -\mathrm{i}[{\hat{H}},{\hat{\rho }}] + \frac{1}{4}\sigma ^2 \left[ {\hat{L}} {\hat{\rho }} {\hat{L}} - \frac{1}{2} \left( {\hat{L}}^2 \rho + {\hat{\rho }} {\hat{L}}^2 \right) \right] \end{aligned}$$

for the reduced density matrix ${\hat{\rho }}$ of the system, so we are left with the signal-processing solution (11) to the stochastic unravelling dynamics, where $\phi _{it}=\sqrt{\pi _{it}}$ and where $\pi _{it}$ is given by (5). Putting it differently, if we define a matrix ${\hat{\rho }}$ by setting its (*i*, *j*) element to be

15 $$\begin{aligned} {\hat{\rho }}_{ij} = \mathrm{e}^{-\mathrm{i}(E_i-E_j)t} \, {\mathbb E}\left[ \sqrt{\pi _{it}\, \pi _{jt}} \right] , \end{aligned}$$

where ${\mathbb E}[-]$ denotes expectation over all random paths $\{\xi _t\}$, and if $\{\pi _{it}\}$ is the solution (5) to the signal detection problem, then the matrix ${\hat{\rho }}$ satisfies the Lindblad equation (14) for the dynamics of the open quantum system. In other words, the density matrix defined by ${\hat{\rho }}={\mathbb E}[ |\psi _t\rangle \langle \psi _t|]$ solves the Lindblad equation (14) when the state vector $|\psi _t\rangle$ is the solution to (12). We remark that here we have restricted our discussion to Lindblad operators that are Hermitian, but an analogous conclusion can be deduced when they are not Hermitian, leading to the so-called quantum filtering equations^24^.

More generally, if $[{\hat{H}},{\hat{L}}]\ne 0$, then we introduce a time-reversed state $|\varphi _t\rangle = \mathrm{e}^{\mathrm{i}{\hat{H}}t} |\psi _t\rangle$, and let ${\hat{L}}_t = \mathrm{e}^{\mathrm{i}{\hat{H}}t} \, {\hat{L}} \, \mathrm{e}^{-\mathrm{i}{\hat{H}}t}$. Then a short calculation shows that the stochastic Schrödinger equation for $|{\varphi _t}\rangle$ is given by

16 $$\begin{aligned} \mathrm{d}|\varphi _t\rangle = \frac{1}{2}\sigma ({\hat{L}}_t-\langle {\hat{L}}_t\rangle ) |\varphi _t\rangle \, {\mathrm{d}}W_t - \textstyle {\frac{1}{8}} \sigma ^2({\hat{L}}_t-\langle {\hat{L}}_t\rangle )^2 |\varphi _t\rangle \, {\mathrm{d}}t , \end{aligned}$$

where $\langle {\hat{L}}_t\rangle = \langle {\varphi }_t| \,{\hat{L}}_t \, |\varphi _t\rangle$. Notice that in the time-reversed representation the unitary term associated with the Hamiltonian is removed from the dynamical equation. Thus, the solution for $|\varphi _t\rangle$ is obtained by finding the signal detection problem for which the time-dependent signal process *L*(*t*) corresponds to the spectrum of the quantum operator $\mathrm{e}^{\mathrm{i}{\hat{H}}t} \, {\hat{L}} \, \mathrm{e}^{-\mathrm{i}{\hat{H}}t}$, and for the (non-Markovian) information process we have

17 $$\begin{aligned} \xi _t = \sigma \int _0^t L(s)\,\mathrm{d}s + B_t . \end{aligned}$$

Specifically, letting $\pi _{it}={\mathbb P}(L(t)=l_i|\{\xi _s\}_{s\le t})$, the solution to (16) can be constructed by setting $|\varphi _t\rangle = \sum _i \sqrt{\pi _{it}} \, \mathrm{e}^{\mathrm{i}\theta _i}\, | l_i\rangle$.

It is worth remarking that a stochastic unravelling of the Lindblad equation for the reduced density matrix of the system in terms of a random pure-state dynamics is by no means unique, and thus is not restricted to the Ito stochastic differential equations of the form (12). Indeed, the underlying noise type need not take the form of a Brownian motion: depending on the context of the experiment the noise type may well be modelled more appropriately from a considerably broader family of processes known as Lévy processes, of which Brownian motion is just one example. However, for each noise type (e.g., a Poisson process, a gamma process, a variance gamma process, and so on) there is a canonical way of formulating the signal detection problem^25^, and deduce the corresponding conditional density process $\{\pi _{it}\}$. Then by defining $\phi _{it}= \sqrt{\pi _{it}}$ and substituting the result in (11) we obtain the corresponding stochastic unravelling of the same Lindblad equation. Hence in each case we are left with the problem of signal processing in one form or another. It is also worth noting that the open quantum dynamics for the density matrix may take a form more general than the familiar Lindblad equation. For example, if the Markovian approximation used to deduce the dynamics of the reduced density matrix is not applicable, then one is lead to a corresponding stochastic unravelling for the wave function evolution that is manifestly non-Markovian^26^. Whether these more general equations admit an explicit signal detection interpretation remains an open question, though we would speculate from the structure of the unravelling equation^26^ that the answer will be affirmative.

At any rate, the foregoing analysis demonstrates that the stochastic unravelling of the Lindblad equation *necessarily takes the form of the optimal signal detection*. In the Brownian case the dynamical equation (16) that gives rise to a seemingly random and yet purposeful evolution is a Hilbert space formulation of the Kushner equation^27,28^ in signal processing. Importantly, the Wiener process $\{W_t\}$ appearing in (16) does not represent noise, but is the innovations process. Hence the evolution of the wave function follows the random path that is determined by the “best” estimate of the underlying signalling problem: It is not the case that the state of an open quantum system is randomly perturbed by means of a noisy Brownian motion (as in the classical Brownian particle). For sure the state is influenced by the noise $\{B_t\}$, but its evolution follows the path determined only by the arrival of new information resulting from the interaction with the environment regarding the stable state of the system, corresponding to the eigenstates of the Lindblad operator. In other words, the wave function of the system is the representation of the conditions of their uncertain environments.

## Quantum dynamics for motion tracking

This feature of quantum dynamics resembles, at least at an intuitive level, the behaviour of biological systems. With this in mind we attempt to model motions of biological systems—plants in this case—in response to changing environments using stochastic Schrödinger equations. To characterise the tracking motion we consider a model of the type discussed, for instance, by Diosi^15^, in which the Lindblad operator is the position operator: ${\hat{L}}=\frac{1}{2}\sigma {\hat{Q}}$, where $\sigma$ is a constant. The effect of the Lindbladean part of the dynamics is to localise the wave function at the hidden “true” value of the position, which in the present context may represent the location of the light source. Plants do not possess sophisticated visual sensors, so a priori the position of the light source is hidden to them. However, they possess a range of photoreceptors to detect and analyse incident light so as to regulate their responses to the environments^29^. From the viewpoint of signal detection, the target—e.g., the position of the sun—is moving, and this will be modelled by choosing the Hamiltonian to be just the momentum operator: ${\hat{H}}= \mu {\hat{P}}$, where $\mu$ is the rate parameter. Thus we have the dynamical equation

18 $$\begin{aligned} \mathrm{d}\psi _t(x) = -\mathrm{i}\mu {\hat{P}} \, \psi _t(x)\, \mathrm{d}t + \textstyle {\frac{1}{2}} \sigma \big ( {\hat{Q}} - \langle {\hat{Q}}\rangle _t \big ) \psi _t(x) \, \mathrm{d}W_t - \textstyle {\frac{1}{8}} \sigma ^2 \big ( {\hat{Q}} - \langle {\hat{Q}}\rangle _t \big )^2 \psi _t(x) \, \mathrm{d}t . \end{aligned}$$

The idea that we propose here therefore is to regard the squared wave function $\pi _t(x)=|\psi _t(x)|^2$, where $\psi _t(x)$ is the solution to (18), as representing the probability distribution of the location of the light source, as “perceived” by the plant (along the east-to-west straight line, which we take to approximate the actual motion of the sun on the upper hemisphere via vertical projection; the model here is understood to capture the qualitative behaviour of motion tracking for illustrative purposes). In other words, the integral $\int x\,\pi _t(x)\,\mathrm{d}x$ represents the best estimate of the location of the light source arrived at by the plant from the data gathered through a range of receptors.

For simplicity let us assume that the initial state is a standard Gaussian state: $\psi _0(x) = (2\pi )^{-1/4} \exp (-\frac{1}{4}x^2)$. The signal detection solution to (18) is then as follows. We let *X* be a standard normal random variable with mean zero and variance one, and consider the “signal” process $X_t = X + \mu t$. The observation of the signal, however, is obscured by a Brownian noise, giving rise to the information process

19 $$\begin{aligned} \xi _t = \sigma \int _0^t X_s \, \mathrm{d}s + B_t. \end{aligned}$$

Then by considering the conditional density ${\mathbb P}(X_t=x|\{\xi _s\}_{s\le t})$ that determines the best estimate for the location of the target we deduce, after a short calculation, that the full solution to (18)—not merely the asymptotic steady-state solution^30,31^—with the Gaussian initial state is given by

20 $$\begin{aligned} \psi _t(x) = \left( \frac{1+\sigma ^2t}{2\pi }\right) ^{\frac{1}{4}} \exp \left( -\frac{\left[ (1+\sigma ^2t)x - \sigma \xi _t - \mu t (1+\frac{1}{2} \sigma ^2 t ) \right] ^2 }{4(1+\sigma ^2t)} \right) , \end{aligned}$$

where the innovations Brownian motion $\{W_t\}$ in (18) is related to the information process $\{\xi _t\}$ according to

21 $$\begin{aligned} \mathrm{d}W_t = \mathrm{d}\xi _t - \sigma \left( \frac{\sigma \xi _t + \mu t (1+\frac{1}{2} \sigma ^2 t)}{1+\sigma ^2t} \right) \mathrm{d}t . \end{aligned}$$

In the present case where the initial state is Gaussian, the best estimate for the position of the light source:

22 $$\begin{aligned} \int _{-\infty }^\infty x\,\pi _t(x)\,\mathrm{d}x = \frac{\sigma \xi _t + \mu t \left( 1+\frac{1}{2} \sigma ^2 t\right) }{1+\sigma ^2t} \end{aligned}$$

is a linear function of the information process $\{\xi _t\}$, which seems reasonable. More generally, in the case of an arbitrary initial state $\psi _0(x)$, writing $p(x)=|\psi _0(x)|^2$, letting *X* be a random variable with the density *p*(*x*), and setting $X_t=X+\mu t$, the solution to (18) is determined by the conditional probability process:

23 $$\begin{aligned} \pi _t(x) = \frac{p(x) \, \exp \left( \sigma (x-\mu t)\left( \xi _t-\frac{1}{2}\sigma xt\right) \right) }{\int p(x) \, \exp \left( \sigma (x-\mu t)\left( \xi _t-\frac{1}{2}\sigma xt\right) \right) \, \mathrm{d}x} . \end{aligned}$$

That is, $\psi _t(x)=\mathrm{e}^{\mathrm{i}\theta (x)}\sqrt{\pi _t(x)}$, where $\theta (x)$ in the initial phase. The relatively simple model constructed here appears effective in characterising the simple solar-tracking motion whereby the plant orients towards the direction of the sun but with small errors. In particular, if the distribution of the error in the orientation of the plant leaves and flowers were normally distributed, then we find that the Gaussian model (22) would be appropriate.

## Quantifying the information extracted

We are interested in the question on how much information extraction is needed so as to deduce the location of the unknown moving target $\{X_t\}$. In a spontaneous localisation model like (18) the large-time behaviour of the system is not physically relevant, for, in the limit $t\rightarrow \infty$ the wave function converges to (the square root of) a delta function at the point corresponding to the true value of *X*, but this requires an infinite amount of information, or, equivalently, infinite reduction in entropy.

In the case of solar tracking of plants, on the other, the sun is not a point particle. Likewise, the leaves or flowers are not positioned perfectly perpendicular to the incident light. Hence the localisation model (18) is only relevant up to the time when a good progress is made in terms of tracking the moving sun. This intuitive idea can be made precise by studying the uncertainty (variance) measure of *X*: A good progress is made if the conditional variance of *X* is reduced to a fraction of the initial variance^21,32,33^. In the present context, this timescale $\tau$ is given by $\tau \sim 1/\sigma ^2 \Delta X^2$, where $\Delta X^2$ is the initial variance of *X*.

To quantify the information extraction, therefore, we consider the reduction $S_0-S_\tau$ of the Shannon–Wiener entropy

24 $$\begin{aligned} S_t = -\int \pi _t(x) \log \pi _t(x) \mathrm{d}x, \end{aligned}$$

where $\pi _t(x)=|\psi _t(x)|^2$. Note that we are not concerned with the von Neumann entropy here, which represents the observer’s knowledge, or lack of it, of the system; whereas the Shannon–Wiener entropy represents the system’s lack of knowledge of the environment. To see this, we recall that density matrix of the system is given by

25 $$\begin{aligned} \rho _t(x,y) = {\mathbb E} \left[ {\overline{\psi _t(x)}} \psi _t(y)\right] , \end{aligned}$$

where ${\mathbb E}$ denotes expectation over all random paths for the information process $\{\xi _t\}$, and it is $\rho _t(x,y)$ that fulfils the deterministic Lindbladean dynamical equation describing the time evolution of the open system. Because an (intelligent) observer is unaware which paths the information process has chosen, but knows only of its statistical distribution, the “state” of the system as perceived by the external observer is given by the density matrix $\rho _t(x,y)$. The von Neumann entropy

26 $$\begin{aligned} -\mathrm{tr}(\rho \ln \rho ) = -\iint \rho _t(x,y)\ln \rho _t(y,x)\, \mathrm{d}y\, \mathrm{d}x \end{aligned}$$

thus represents the observer’s lack of knowledge of the exact state of the system. In contrast, since $\pi _t(x)=|\psi _t(x)|^2$ represents the (unintelligent) system’s knowledge of the environment, gathered by optimally processing the noisy information about the environment, it is the Shannon–Wiener entropy that represents the system’s lack of knowledge of the environment.

Because the entropy process $\{S_t\}$ is stochastic, and depends on the information process $\{\xi _t\}$, it can increase or decrease, but on average it decreases^21^. Hence we are interested in the averaged entropy reduction $\Delta S =S_0-{\mathbb E}[S_\tau ]$ for the information gain. In the special case where the initial state is Gaussian, however, the associated entropy is deterministic and is given by

27 $$\begin{aligned} S_t = \frac{1}{2}\left[ 1+\log (2\pi ) - \log (1+\sigma ^2t)\right] , \end{aligned}$$

from which it follows that $\Delta S = \frac{1}{2}\log (1+\sigma ^2\tau )$. On the other hand the initial uncertainty of *X* in this example is unity so that $\tau =\sigma ^{-2}$. Hence $\Delta S = \frac{1}{2}\log 2$ and we find that the amount of information processed to track the motion is half of one bit. Allowing for a variability in the initial wave function, we thus conclude that the amount of information processed to track the motion is at most of order few bits.

## Information erasure and heat production

It seems reasonable to assume that much of the information processed for plant movements are not stored in the plant indefinitely. (Note that this need not apply universally. For instance, there are suggestions that Mimosa plants can be trained to learn certain behaviours, which it can remember for more than a month^34^.) But the loss of processed information has to be distinguished from information that are encoded in their genes. Take, for instance, the seed of a plant buried in dark soil. Information encoded in the seed cells sends roots towards the direction of the gravitational pull, and stems in the opposite direction—the so-called gravitropism. However, plants a priori do not possess information about their environments, so measurements are performed to detect the direction of the gravitational field. In particular, if the direction of the gravitational force changes, then roots will change the orientation of their growths to accommodate this change of the environment^35^.

We are not concerned here with the actual mechanism by which plans detect gravitational field, which has only been unravelled relatively recently^13,14^. What concerns us is the information-processing aspect of the growth orientation selection, and this can be modelled heuristically using a stochastic Lindbladean dynamical equation for a two-level system with ${\hat{H}},\,{\hat{L}}\propto {\hat{\sigma }}_z$; the solution to which in the quantum context are known^16,21^. Specifically, we can think of a uniform magnetic field in the vertical *z*-direction surrounding a spin-$\frac{1}{2}$ particle in quantum mechanics as the analogue of the gravitational field surrounding the seed in biology. The direction of the spin then represents the orientation of the root growth. Under the dynamics governed by such stochastic Schrödinger equation the spin of the particle, which initially could be pointing in any orientation, will follow a random path such that eventually it will line up parallel to the direction of the field, and this reorientation of spin can be interpreted as modelling the root reorientation in gravitropism. Thus in this model the information process is $\xi _t=\sigma L t + B_t$, where the random variable *L* takes the value $+1$ with probability $p=|\langle \uparrow \!|\psi _0\rangle |^2$, and takes the value $-1$ with probability $1-p=|\langle \downarrow \!|\psi _0\rangle |^2$. Here, $|\psi _0\rangle$ is the initial wave function of the system, and $(|\!\uparrow \rangle ,|\!\downarrow \rangle )$ are the two eigenstates of the Pauli matrix ${\hat{\sigma }}_z$, corresponding to the spin-up and spin-down states. The effect of the Hamiltonian ${\hat{H}}=g{\hat{\sigma }}_z$ is then to generate the nutation of the root with angular frequency determined by the parameter *g*, while the effect of the Lindblad operator ${\hat{L}}=\gamma {\hat{\sigma }}_z$ is to generate gravitropism at a rate that is governed by the parameter $\gamma$.

Because the problem here is of binary nature, of order one bit of information is processed to decide the growth orientation. This information is transient; measurements are performed repeatedly (by continuous monitoring) to reaffirm the orientation of the gravitational field^7^. Thus, one bit of information must be regularly erased from each information-bearing unit inside the cells; most likely in the elongation zone cells, for, it seems implausible that such information is transmitted and stored elsewhere in the plant, because this will require additional resource for error corrections.

Returning to heliotropism, take, for instance, the case of sunflowers. While mature sunflowers are permanently facing east, young sunflowers trace the motion of the sun. After sunset, the flowers then turn around from west to east and await the sunrise. This indicates a memory effect. Indeed, if one were to rotate a pot of a young sunflower plant by, say, $\pi /2$, then the sunflower now turns back and forth between north and south for a few days before stopping^11^. In other words, the memory is lost only after a while. According to our rough estimate above, this results in the erasure of at most few bits of information. On the other hand, in the case of a daisy flower, overnight the flower tend to orient in a random direction, indicating a faster loss of information.

It is known that information erasure is necessarily accompanied by energy consumption and hence heat production, resulting in an increase of environmental entropy^17^. In particular, the minimum amount of energy consumption required for the erasure of one bit of information at temperature *T* is $k_\mathrm{B}T\log 2$. Based on our estimate, a daisy flower at summer-night temperature (say, 290 K) will thus consume at least of order $10^{-2}$ eV of energy per each information-encoding unit. This in turn results in heat production. A detection of this effect is likely to be difficult, for, the magnitude of energy involved for information erasure based on our estimate is likely to be rather small compared to that for plant movements. Further, the environment of plants are far removed from the highly controlled environment in which such an effect has been detected in a quantum system^36^.

The precise mechanism of how plants store information is not fully understood. Hence it is not known how many information-storing units are contained in a given plant, making it difficult to estimate the total energy cost of information erasure. To get a better intuition for the scale involved, therefore, let us consider, as an example, the circumnutation of a common bean plant^5^ by assuming that each cell contains at most a single such unit. For the bean plant to identify the location of an object like a pole to climb up within a $\pm \,10^\circ$ angular window with $95\%$ confidence, say in a recent experimental setup^5^, it must process between 15 and 20 bits of information. This estimate follows from the assumption that the a priori probability of finding an object is uniform over the circle around the plant. Assuming that the a posteriori distribution can be described by any one of the standard circular distributions such as the von Mises or the wrapped normal distribution, one arrives at this estimate. Now if the object is removed, this information must be erased. If the plant can process information relatively close to the Landauer limit, say, $10^4$ times the limit, then the cost of erasure is of order $10^{4}$ eV, which would be approximately just below $1\%$ of the total energy consumed by the cell^37^. Intuitively this is a plausible figure, given the limited energy resource available to biological systems. Or, putting the matter differently, if information is processed significantly less efficiently, say at the level of our everyday computers, then the erasure cost becomes too high for survival. Our hypothesis that biological systems can process information close to the Landauer limit is consistent with the empirical observation that plants produce very little heat (except for thermogenic plants that purposefully produce heat^38^).

## Second law in biology

In accordance with the Landauer principle the erasure of information will increase the entropy of the environment. It is tempting therefore to conjecture that biological systems operate by extracting information from their environments, processing them, and arriving at the best estimate of the state of the environment for the purpose of adaptation. This will result in the gain of information, and thus reduction in entropy. However, some, or much of the processed information is lost, resulting in increasing entropy. The process of information erasure then must lie at the heart of ageing—or arrow of time—in biological systems.

It should be added that while the present paper concerns the dynamical mechanism of the information-processing of the environmental conditions of biological systems, our estimates show that the erasure cost, and hence entropy production, of genetic information encoded a priori in biological systems would be substantially more significant than that of the a posteriori processed information. This is consistent with the empirical fact that the life span of a biological system on average reduces when genetic information encoded in the system is lost. Hence in terms of biological arrow of time, we argue that the erasure of processed information will not constitute the dominant contributing factor.

## Model calibration

**Figure 1:**
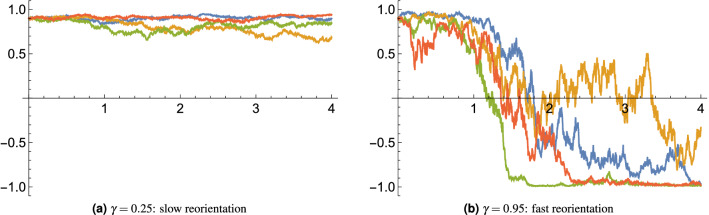
Model simulation of the binary root reorientation. In a model with ${\hat{L}}=\gamma {\hat{\sigma }}_z$, assume that the plant is initially 95% confident that the gravitational force is pointing down (the signal $L=-1$); but at time zero it is flipped upside-down (the signal $L=+1$). Depending on the value of the parameter $\gamma$ the reorientation (and hence the loss of the initial memory) can be slow or fast. Four sample paths for the order parameter $\langle {\hat{\sigma }}_z\rangle _t$ are shown here each for the two chosen values of $\gamma$. The order parameter represents the vertical component of the unit directional vector of the root. Because the reorientation timescale is proportional to $\gamma ^{-2}$, although in all cases the root will eventually point down, this process can take a long time if $\gamma$ is small (the left panel); whereas for a larger value of $\gamma$ the reorientation takes place rapidly (the right panel).

How can model parameters be estimated against data? In our simple tracking model we have the rate parameter $\sigma$, which determines the timescale that plants can orient towards the light source. For instance, leaves of Cornish mallow can reorient as rapidly as $2\pi /9$ per hour^39^, indicating that $\sigma$ takes a large value. (Recall that the response timescale is inverse proportional to $\sigma ^2$ so that a rapid response would imply a large $\sigma$.) If plants exhibit a memory effect, like circadian rhythm of sunflowers^11^, then either the variance of the signal is small or else the value of $\sigma$ is small. In the case of gravitropism, using the model described above, if we let ${\hat{L}}=\gamma {\hat{\sigma }}_z$, then the parameter $\gamma$ can be calibrated from studying the timescale of root reorientation by turning the growing plant still in the soil upside-down. Figure 1 shows how the response time of the plant can be slow or fast, depending on the value of $\gamma$.

## Discussion

In summary, we have shown how stochastic unravelling of the Lindblad equation for open system dynamics in quantum mechanics necessarily takes the form of the equation for the best information processing in signal detection. This observation suggests that the notion of an optimal information-processing capability is fundamental to the laws of nature at the quantum level. Such a statement may at first seem counterintuitive, for, one tends to associate some form of intelligence to the concept of information processing. In physics one is more accustomed to the idea of a variational principle to arrive at laws of nature. However, our conclusion can in fact be reached by means of a variational argument, albeit in noisy environments. Namely, the dynamical equation for the state of the system can be derived by demanding that the average entropy reduction of the system is maximised. Indeed, the entropy and variance—the two uncertainty measures—are closely related in the context of signal processing. This follows from the fact^21^ that the Shannon–Wiener entropy associated with the stochastic Schrödinger equation (12) satisfies the relation

28 $$\begin{aligned} {\mathbb E}\left[ S_\tau \right] = \frac{1}{2}\sigma ^2 \int _\tau ^\infty {\mathbb E}\big [\Delta L_t^2\big ]\, \mathrm{d}t , \end{aligned}$$

where $\Delta L_t^2=\langle \psi _t| ({\hat{L}}-\langle {\hat{L}}\rangle _t)^2 |\psi _t\rangle$ is the conditional variance of the Lindblad operator. Hence minimisation of the quadratic error is linked to maximisation of the entropy reduction.

Given that biological systems like plants use sophisticated mechanisms to process external signals concerning light, water, temperature, gravity, organic compounds, and so on, and adapt their behaviours accordingly, we postulated that the stochastic unravelling of the Lindblad equation in open quantum systems can be applied to model the dynamical behaviours of plants in open environments. We considered a localisation model to capture qualitatively the tracking motion, to arrive at a rough estimate of the quantity of information processed. The idea that information processing in biological systems must be viewed as fundamental has been advocated before^40–42^, but here we have been able to quantify this in a dynamical context. Empirical observations suggest that some of the processed information is erased, from which we postulated (a) that biological systems must operate relatively close to the Landauer limit of computation; and (b) that information erasure must underlie the ageing of biological systems in a fundamental way.

It is worth remarking that the specific models of stochastic Schrödinger equations considered above for the characterisation of plant motions can be presented by use of purely classical signal detection techniques. While this is consistent with our thesis that signal processing capability is fundamental to laws of nature including quantum theory, it also implies that the resulting estimates in the context of biology, at least in the examples considered here, in principle could have been obtained purely in terms of classical signal detection, without referencing quantum theory. That said, it would be technically more challenging to pose a model for the spherical nutation of roots using a purely classical language, whereas our quantum spin-$\frac{1}{2}$ model offers a highly effective and simple treatment of the matter. More importantly, we believe that in many realistic setups in biology, more adequate descriptions can be obtained by means of more quantum-mechanical models for which the commutator of the Hamiltonian ${\hat{H}}$ and the Lindblad operator ${\hat{L}}$ is nontrivial (that is, $[{\hat{H}},{\hat{L}}]$ neither vanishes nor is proportional to the identity). In the biological context, the Lindblad operator represents adaptation, while the Hamiltonian represents changes of the environment. (For example, in the simple motion-tracking model considered here, the Lindblad operator has the effect of orienting towards the location of the sun, whereas the Hamiltonian changes the location of the sun.) It is inadvertently the case in biology and ecology that adaptation is possible so long as environmental changes are sufficiently slow, whereas a fast change can be catastrophic to the survival of the biological system, leading to a very different dynamical behaviour. This is already seen even in the simple phenomenon of heliotropism^6^. Such a transition can be described by a stochastic Schrödinger equation for which $[{\hat{H}},{\hat{L}}]\ne 0$ in a nontrivial way, because in such a model one typically encounters a phase transition in the dynamical behaviour of the system^43,44^, depending on the relative strengths of ${\hat{H}}$ and ${\hat{L}}$. Conversely, without the lack of commutativity it is not possible to describe such critical phenomena seen in biology and ecology; yet, the notion of incompatible observables is one of the signatures of quantum theory. We argue that this observation justifies our unified approach to model dynamical behaviours of quantum and biological systems in open environments, and hope to develop more general theories elsewhere.

We conclude by remarking that there is an ongoing debate in plant science concerning the notion of consciousness and sentience in plants^45–48^. This follows from advances in detecting plants’ remarkable abilities in observing, analysing, and adapting to the changing environments that surround them. Our postulate that information-processing capability is part of the laws of nature, thus not requiring any intelligence, might shed a new light on this debate.

## References

[1] Nakagaki T, Yamada H, Tóth Á (2010). Maze-solving by an amoeboid organism. Nature.

[2] Tero A (2010). Rules for biologically inspired adaptive network design. Science.

[3] Gruntman M, Novoplansky A (2004). Ohysiologically mediated self/non-self discrimination in roots. Proc. Natl. Acad. Sci. USA.

[4] Runyon JB, Mescher MC, De Moraes CM (2006). Volatile chemical cues guide host location and host selection by parasitic plants. Science.

[5] Raja V, Silva PL, Holghoomi R, Calvo P (2020). The dynamics of plant nutation. Sci. Rep..

[6] Vandenbrink JP, Brown EA, Harmer SL, Blackman BK (2014). Turning heads: The biology of solar tracking in sunflower. Plant Sci..

[7] Sato EM, Hijazi H, Bennett MJ, Vissenberg K, Swarup R (2014). New insights into root gravitropic signalling. J. Exp. Bot..

[8] Darwin CR, Darwin F (1880). The Power of Movement in Plants.

[9] Bose, J. C. *The motor mechanism of plants*. (London: Longmans, Green and Co., 1928)

[10] Yin HC (1938). Diaphototropic movement of the leaves of Malva neglecta. Am. J. Bot..

[11] Atamian HS, Creux NM, Brown EA, Garner AG, Blackman BK, Harmer SL (2016). Circadian regulation of sunflower heliotropism, floral orientation, and pollinator visits. Science.

[12] Goyal A, Szarzynska B, Fankhauser C (2013). Phototropism: At the crossroads of light-signaling pathways. Trends Plant Sci..

[13] Baluška F, Mancuso S (2013). Root apex transition zone as oscillatory zone. Front. Plant Sci..

[14] Vandenbrink JP, Kiss JZ (2019). Plant responses to gravity. Semin. Cell Dev. Biol..

[15] Diósi L (1988). Continuous quantum measurement and Ito formalism. Phys. Lett. A.

[16] Brody DC, Hughston LP (2002). Efficient simulation of quantum state reduction. J. Math. Phys..

[17] Leff HS, Rex AF (2003). Maxwell’s Demon 2.

[18] Patten BC (1959). An introduction to the cybernetics of the ecosystem: The trophic-dynamic aspect. Ecology.

[19] Trewavas, A. Information, noise and communication: Thresholds as controlling elements in development. In *Biocommunication of Plants* (G. Witzany & F. Baluška, eds) (Berlin: Springer-Verlag, 2012).

[20] Belavkin VP, Staszewski P (1992). Nondemolition observation of a free quantum particle. Phys. Rev. A.

[21] Brody DC, Hughston LP (2006). Quantum noise and stochastic reduction. J. Phys. A.

[22] Brody, D. C. and Hughston, L. P. Quantum state reduction. In *Collapse of the Wave Function: Models, Ontology, Origin, and Implications*. S. Gao, ed. (Cambridge: Cambridge University Press, 2018).

[23] Kailath T (1968). An innovations approach to least squares estimation, part I: Linear filtering with additive white noise. IEEE Trans. Autom. Control.

[24] Gough JE (2018). The Gisin–Percival stochastic Schrödinger equation from standard quantum filtering theory. J. Math. Phys..

[25] Brody DC, Hughston LP, Yang X (2013). Signal processing with Lévy information. Proc. R. Soc. Lond. A.

[26] Diósi L, Gisin N, Strunz WT (1998). Non-Markovian quantum state diffusion. Phys. Rev. A.

[27] Kushner HJ (1964). On the differential equations satisfied by conditional probability densities of Markov processes, with applications. J. Soc. Ind. Appl. Math. Control A.

[28] Bucy RS (1965). Nonlinear filtering theory. IEEE Trans. Autom. Control.

[29] Paik I, Huq E (2019). Plant photoreceptors: Multi-functional sensory proteins and their signaling networks. Semin. Cell Dev. Biol..

[30] Ghirardi GC, Rimini A, Weber T (1986). Unified dynamics for microscopic and macroscopic systems. Phys. Rev. D.

[31] Diósi L (1988). Localized solution of a simple nonlinear quantum Langevin equation. Phys. Lett. A.

[32] Hughston LP (1996). Geometry of stochastic state vector reduction. Proc. R. Soc. Lond. A.

[33] Adler SL (2002). Environmental influence on the measurement process in stochastic reduction models. J. Phys. A.

[34] Gagliano M, Renton M, Depczynski M, Mancuso S (2014). Experience teaches plants to learn faster and forget slower in environments where it matters. Oecologia.

[35] Knight TA (1806). On the direction of the radicle and germen during the vegetation of seeds. Philos. Trans. R. Soc. Lond..

[36] Bérut A, Arakelyan A, Petrosyan A, Ciliberto S, Dillenschneider R, Lutz E (2012). Experimental verification of Landauer’s principle linking information and thermodynamics. Nature.

[37] Milo R, Phillips R (2016). Cell Biology by the Numbers.

[38] Watling JR, Grant NM, Miller RE, Robinson SA (2008). Mechanisms of thermoregulation in plants. Plant Signal. Behav..

[39] Koller D, Shak T, Briggs WR (1990). Enhanced diaphototropic response to vectorial excitation in solar-tracking leaves of Lavatera cretica by an immediately preceding opposite vectorial excitation. J. Plant Physiol..

[40] Dancoff SM, Quastler H, Quastler H (1953). The information content and error rate of living things. Information Theory in Biology.

[41] Binder PM, Danchin A (2011). Life’s demons: Information and order in biology. Eur. Mol. Biol. Organ. Rep..

[42] Mescher MC, Pearse IS (2016). Communicative interactions involving plants: Information, evolution, and ecology. Curr. Opin. Plant Biol..

[43] Bassi A, Ippoliti E (2004). Numerical analysis of a spontaneous collapse model for a two-level system. Phys. Rev. A.

[44] Brody DC, Longstaff B (2019). Evolution speed of open quantum dynamics. Phys. Rev. Res..

[45] Baluška F, Reber A (2019). Sentience and consciousness in single cells: How the first minds emerged in unicellular species. Bio Essays.

[46] Taiz L, Alkon D, Draguhn A, Murphy A, Blatt M, Hawes C, Thiel G, Robinson DG (2019). Plants neither possess nor require consciousness. Trends Plant Sci..

[47] Trewavas A, Baluška F, Mancuso S, Calvo P (2020). Consciousness facilitates plant behavior. Trends Plant Sci..

[48] Calvo P, Baluška F, Trewavas A (2021). Integrated information as a possible basis for plant consciousness. Biochem. Biophys. Res. Commun..

